# A prebiotic intervention study in children with autism spectrum disorders (ASDs)

**DOI:** 10.1186/s40168-018-0523-3

**Published:** 2018-08-02

**Authors:** Roberta Grimaldi, Glenn R. Gibson, Jelena Vulevic, Natasa Giallourou, Josué L. Castro-Mejía, Lars H. Hansen, E. Leigh Gibson, Dennis S. Nielsen, Adele Costabile

**Affiliations:** 1 Department of Food and Nutritional Sciences, University of Reading, Reading, RG66AP UK; 2 Clasado Research Services Ltd., Thames Valley Science Park, Reading, RG29LH UK; 3 Division of Computational and Systems Medicine, Imperial College London, London, SW7 2AZ UK; 4 Department of Food Science, Faculty of Science, Food Microbiology, University of Copenhagen, Copenhagen, Denmark; 5 Department of Environmental Science, Aarhus University, Roskilde, Denmark; 6 Health Sciences Research Centre, Life Sciences Department, Whitelands College, University of Roehampton, London, SW15 4JD UK

**Keywords:** Autism, GOS, Microbiota, Prebiotics, Gut symptoms, Sequencing, ^1^H-NMR

## Abstract

**Background:**

Different dietary approaches, such as gluten and casein free diets, or the use of probiotics and prebiotics have been suggested in autistic spectrum disorders in order to reduce gastrointestinal (GI) disturbances. GI symptoms are of particular interest in this population due to prevalence and correlation with the severity of behavioural traits. Nowadays, there is lack of strong evidence about the effect of dietary interventions on these problems, particularly prebiotics. Therefore, we assessed the impact of exclusion diets and a 6-week Bimuno® galactooligosaccharide (B-GOS®) prebiotic intervention in 30 autistic children.

**Results:**

The results showed that children on exclusion diets reported significantly lower scores of abdominal pain and bowel movement, as well as lower abundance of *Bifidobacterium* spp. and Veillonellaceae family, but higher presence of *Faecalibacterium prausnitzii* and *Bacteroides* spp. In addition, significant correlations were found between bacterial populations and faecal amino acids in this group, compared to children following an unrestricted diet. Following B-GOS® intervention, we observed improvements in anti-social behaviour, significant increase of Lachnospiraceae family, and significant changes in faecal and urine metabolites.

**Conclusions:**

To our knowledge, this is the first study where the effect of exclusion diets and prebiotics has been evaluated in autism, showing potential beneficial effects. A combined dietary approach resulted in significant changes in gut microbiota composition and metabolism suggesting that multiple interventions might be more relevant for the improvement of these aspects as well as psychological traits.

**Trial registration:**

NCT02720900; registered in November 2015.

**Electronic supplementary material:**

The online version of this article (10.1186/s40168-018-0523-3) contains supplementary material, which is available to authorized users.

## Background

Autism is a neurodevelopmental disorder characterised by impaired social interaction, verbal and non-verbal communication, and repetitive behaviour. In addition to cognitive aspects, autistic spectrum disorder (ASD) individuals can suffer from gastrointestinal (GI) problems such as abdominal discomfort, pain and gas distension [1]. Causes of these gut difficulties are unknown but have been suggested to involve gut microbiota, in particular reduced number of bifidobacteria and increased *Clostridium* spp., *Desulfovibrio* spp., *Sutterella* spp. and/or Veillonellaceae [2]; altered dietary intake and increased gut permeability [3]. Previous studies reported alterations in gut barrier function and GI issues in ASD individuals [4] with the latter often associated with symptom severity [5]. Adam and colleagues looked at GI dysfunction as a parameter and noticed a strong correlation between GI symptoms and severity of autism [6]. These results were also confirmed by Tomova et al. in a more recent study [7].

Exclusion approaches, such as gluten and casein-free diets (GFCF), have been suggested for their potential benefits, but to date strong empirical evidence of their effect on gut health is lacking.

Observational studies reported alleviation of GI problems and/or improved behavioural traits with GFCF, but associations between restricted diet and symptoms are not always apparent [8–10]. Some studies evaluating mood and behaviour showed significant improvements in behavioural traits [11, 12], while others reported no differences after treatment [13–15].

Human gut microbiota (GM) impacts health and well-being and is known to be strongly influenced by diet [16]. A few studies have focused on GM modulators, such as probiotics in ASDs, but there is inconsistency in the parameters evaluated [17, 18]. Parracho et al. focused on behavioural features, reporting improvement after *Lactobacillus plantarum* WCSF1 administration in ASD children [19], whereas Adam et al. showed significant positive differences in faecal organic acid levels in ASD individuals taking probiotics [6]. Metabolic changes were also observed by Kaluzna-Czaplinska and Błaszczyk [20] who, after 2 months of oral supplementation with *L*. *acidophilus* (strain Rosell-11), found a significant decrease in the level of d-arabinitol (DA)-positive modification cosidering its association with pathogenic *Candida* spp. [20].

Further, Tomova and colleagues evaluated the impact of 4 months of mixed probiotic administration (Children Dophilus) on GM composition in ASD children and they were able to show modulation of the *Bacteroidetes/Firmicutes* ratio and an increase in bifidobacterial numbers [7].

Prebiotics are food ingredients selectively metabolised by indigenous beneficial bacteria thereby positively modulating GM. Their effects in autism are not well documented. Previously, we showed that the prebiotic B-GOS® (a galactooligosaccharide) had an impact on the faecal microbiota composition and metabolic profile using an in vitro fermentation system mimicking conditions of the autistic colon [21]. Its impact in vivo, especially taking into consideration the different dietary approaches that ASD children might follow, has not been investigated. Thus, the purpose of this study was to understand the impact of diet on GM composition and metabolism in ASD children and to investigate the modulating potential of B-GOS® intervention on these paramenters. Additionally, the effect of B-GOS® was evaluated on GI dysfunction, mood, behaviour, and sleep. To our knowledge, this is the first study to evaluate prebiotic potential in ASD.

## Results

### Baseline dietary intake

Food diaries (*N* = 30) were analysed at baseline (before starting with the prebiotic intervention) by comparing daily macronutrient and micronutrient intakes according to different diets that the children were following (exclusion and unrestricted diet). Significant differences were seen only in vitamin D intake (Table 1). ASD children who were on the unrestricted diet had significantly lower vitamin D consumption compared to ASD children on exclusion diets (*P* < 0.01). In addition, vitamin D intake was much lower, for both groups, than daily UK government recommendations (10 μg/day) [19].

**Table 1:** Energy and nutrient intake in children on exclusion and un-restricted diets and comparison with the UK government recommendations for typically developing children

		Exclusion diet		Un-restricted diet		Typically developed children
Daily dietary composition		Mean	SD	Mean	SD	Adequate intake
Energy Intake	Kcal	1579.18	394.19	1478	578.70	1430–1920
Protein Intake	g	56.93	17.30	55.01	15.68	19.7–42.1
Carbohydrate	g	183.58	35.20	187.26	74.99	191–333
Total sugars	g	63.32	27.58	81.46	36.44	19–33
Fibres	g	17.19	5.60	15.36	9.26	17.5–25
Saturated fatty acid	g	22.22	12.26	22.28	12.25	17.5–31
PUFA	g	12.41	6.39	9.88	7.97	10.5–18
MUFA	g	22.88	18.19	19.39	16.76	20.5–36
Vitamin C	mg	73.64	44.46	70.94	68.00	30–35
Vitamin D	μg	2.72	1.40	1.21^**^	1.18	10
Vitamin B1	mg	1.38	0.49	1.38	0.66	0.6–1
Vitamin B2	mg	1.44	0.56	1.41	0.64	0.8–1.2
Vitamin B6	mg	1.15	0.33	1.13	0.53	0.9–1.2
Vitamin B12	μg	3.04	1.84	3.82	2.56	0.8–1.2
Iron	mg	10.37	4.30	8.04	2.99	6.1–11.3
Calcium	mg	536.00	247.89	697.17	357.52	450–1000

*Mean* average of four consecutive days, *SD* standard deviation

**P < 0.01

### GI symptoms and sleep diaries

**Fig. 1:**
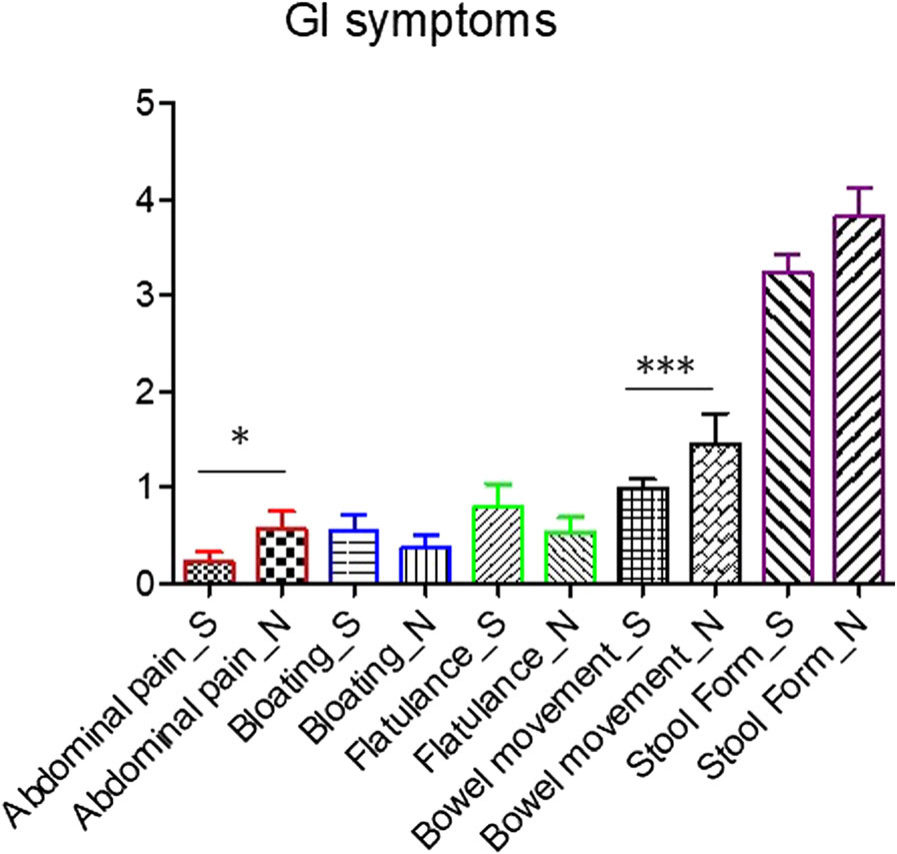
GI symptom assessment during 3 weeks baseline data collection. *S* exclusion diets; *N* unrestricted diet; *P < 0.05; ***P < 0.001

At baseline, scores extracted from GI symptom diaries (*N* = 30) showed that exclusion diets had a significant impact on gastrointestinal problems (Fig. 1). Significantly lower scores of abdominal pain (*P* < 0.05) and bowel movement (*P* < 0.001) were reported in children following exclusion diets.

GI symptoms were also quantitatively evaluated during the intervention period (*N* = 26), and a general trend of reduction in GI problems was reported after B-GOS® use (data not shown), but differences between treatments were not significant. Significant effects were observed for the interaction between diet and time, for bowel movements (*P* < 0.01) and flatulence (*P* < 0.05). Qualitative analysis performed on sleep habits showed that 23% of participants (two ASD following unrestricted diet and one under exclusion diet) benefited from B-GOS® intervention (*N* = 13). Parents reported that their children slept 1 h longer than usual and noticed that the children had less problems falling asleep.

### Anxiety and ASD-related behaviour questionnaires

**Fig. 2:**
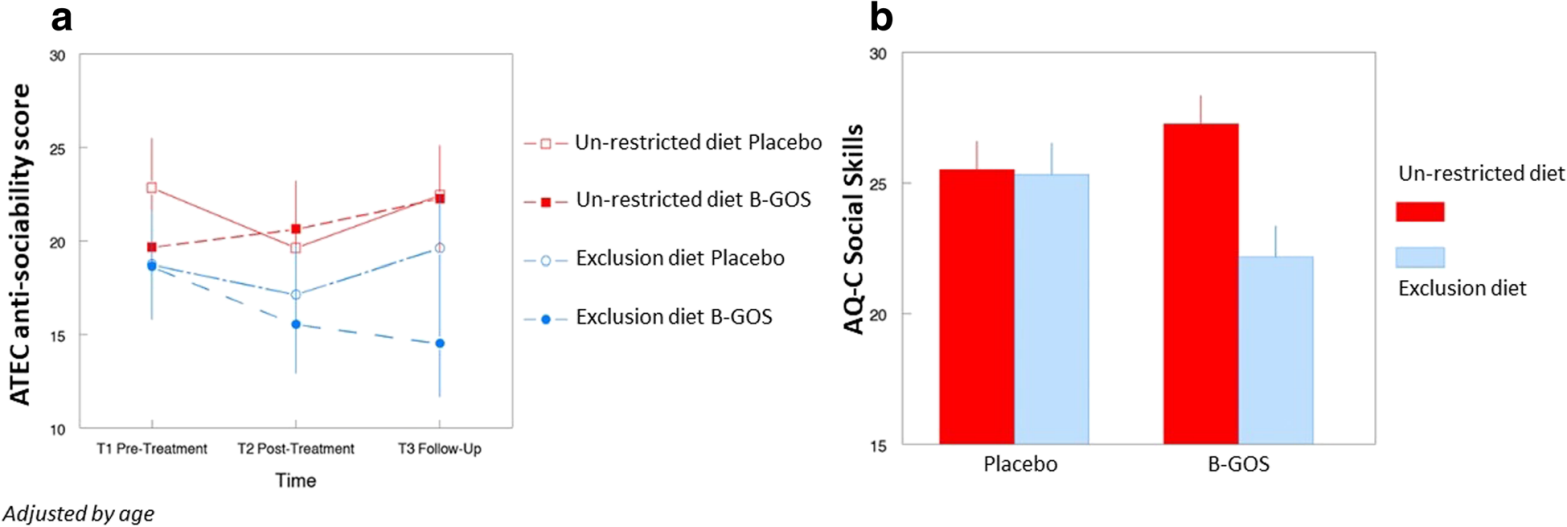
**a** ATEC questionnaire. Results showed consistent reduction over time in anti-sociability score in children on the combination of the exclusion diet and B-GOS intervention, with the most apparent difference occurring at follow-up (time × diet × treatment interaction, *p* = 0.05; adjusted for age). Placebo:Maltodextrin. **b** AQ questionnaire. After intervention and including follow-up, social skills were improved (i.e. scores were lower) by B-GOS treatment in children on the exclusion diet only (diet × treatment interaction, *P* < 0.05). Results were reported as estimated marginal means ± standard error (SE). NB, post-hoc comparisons are not valid where covariates are included

Anxiety and ASD-related behaviour questionnaires (*N* = 26) were analysed taking into account age, diet and intervention. The results showed a significant improvement in social behaviour scores (i.e. scores were lower) after B-GOS® intervention in ASD children following exclusion diets (*N* = 6). Specifically, results from the AQ questionnaire social skills scale (RMANCOVA of T2 and T3 adjusting for T1, diet by treatment interaction, F(1,21) = 4.62, *p* < 0.05) mirror improvements in anti-social behaviour from the ATEC questionnaire (RMANCOVA, adjusting for age, time by diet by treatment interaction, F(2,42) = 3.20, *p* = 0.05). This shows that this aspect of autistic behaviour varied over time depending on both exclusion diet and treatment (Fig. 2a, b). No other behavioural measures were significantly affected.

### Bacterial composition by FISH analysis

FISH analysis was performed on total bacteria and bifidobacteria of the faecal samples collected at baseline, after intervention and at follow-up (*N* = 78). Despite an increase in the number of *Bifidobacterium* spp. after B-GOS intervention (data not shown), FISH results did not show any significance difference between treatments and the interaction between treatments versus diet.

### Gut microbiota composition as determined by 16S rRNA gene amplicon sequencing

#### Baseline

**Fig. 3:**
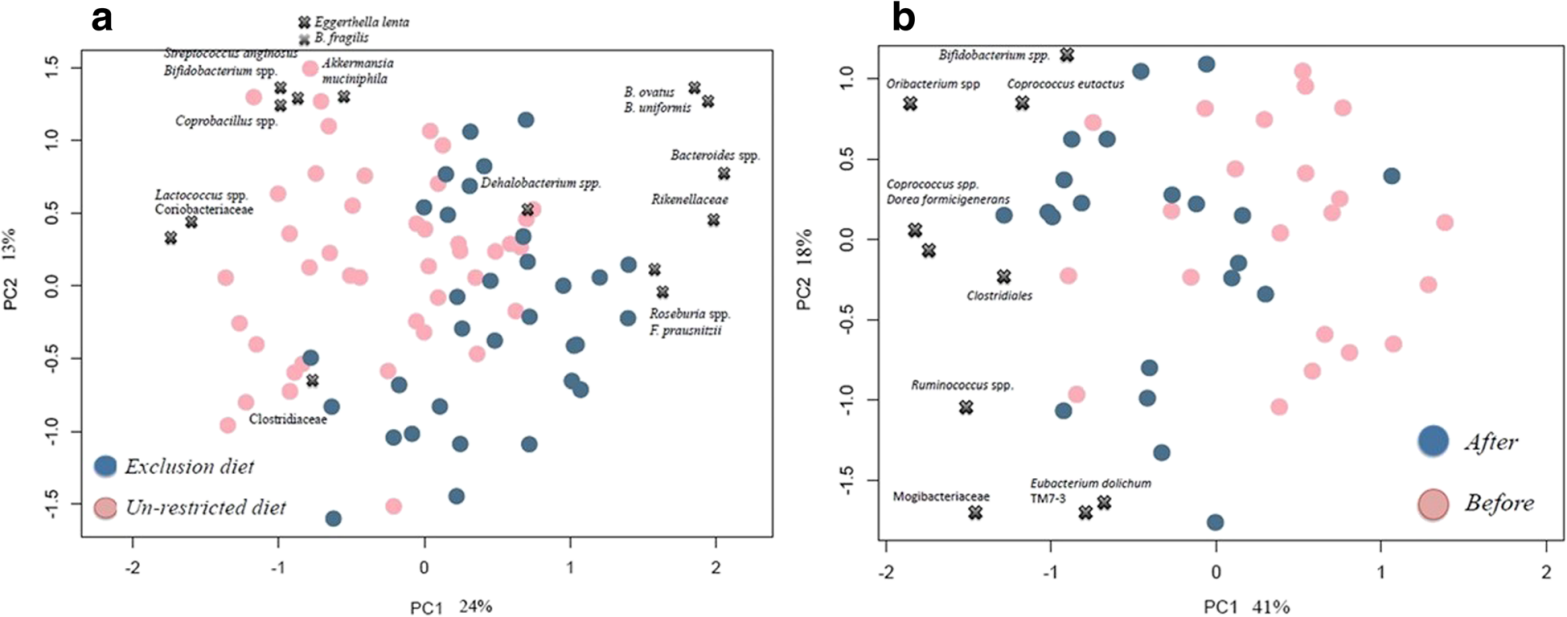
**a** PCA plot showing differences in microbial genera based on diet as determined by RDA analysis. **b** PCA plot displaying differences in microbial genera after B-GOS treatment in un-restricted diet group as determined by RDA analysis. Blue dots: after B-GOS® intervention; pink dots: before B-GOS® intervention. X displays the loading positions of the most discriminative bacterial genera

Søresen-Dice distance based principal coordinate analysis (PCoA, Additional file 1: Figure S1A) showed separation between volunteers (*N* = 30) on exclusion and unrestricted diets at baseline (week 1 and week 2) considering the presence or absence of particular bacteria (*N* = 60 samples). Such separation was not observed on Bray-curtis distance-based PCoA (Additional file 1: Figure S1B) when taking also the relative abundance of GM into consideration, indicating that the main GM differences were driven by variations in the lower abundant bacterial groups. A redundancy analysis (RDA) model was built to assess the impact of the variable ‘diets’ on GM composition (*P* < 0.004). PCA (principal component analysis) biplot showed different bacterial groups significantly associated with separation (Fig. 3a). *Bacteroides* spp. (Bacteroidaceae), Rikenellaceae, *Roseburia* spp. (Lachnospiraceae), *F*. *prausnitzii* (Ruminococcaceae) and Clostridiaceae were present in higher proportion in the exclusion diet group, whereas *Eggerthella lenta*, *Bifidobacterium* spp. (Coriobaceriaceae), *B*. *fragilis* (Bacteroidaceae), *Akkermansia muciphila* (Verrucomicrobiacea), *Streptococcus anginosus*, *Lactococcus* spp. (Streptococcaceae), and *Dehalobacterium* spp. (Dehalobacteriaceae) were present in higher relative abundance in the unrestricted diet (Additional file 2: Table S1). Additionally, bifidobacteria were found in lower abundances (3.5%; log_10_ = 8.95 CFU/g) in the exclusion diet group compared to the unrestricted diet group (4.5%; log_10_ = 9.59 CFU/g). Also, a reduction in the Veillonellaceae family was observed.

#### B-GOS® intervention

A significant RDA model (4% variance; *P* < 0.038) was identified comparing GM composition from ASD children with unrestricted diets, before (baseline; *N* = 21 samples) and after B-GOS administration (*N* = 21 samples). Figure 3b shows PCA biplot reporting bacterial populations positively associated with B-GOS supplementation in ASD children on the unrestricted diet. These included *Bifidobacterium* spp., *Ruminococcus* spp., Lachnospiraceae family (*Coprococcus* spp., *Dorea formicigenerans*, *Oribacterium* spp.), *Eubacterium dolchum*, TM7–3 family and Mogibacteriaceae. Additionally, in this intervention group, rarefaction curves showed that B-GOS® supplementation increased diversity in GM composition, but this increase was not significant (Additional file 3: Figure S2).

Significant results (RDA model; 6% variance; *P* < 0.008) were identified comparing the effect of treatments (placebo vs B-GOS®) on gut microbiota composition of ASD children following exclusion diet after 6 weeks intervention. Additional file 4: Figure S3 shows different GM profile between the two groups analysed (Additional file 4: Figure S3A) and the main bacterial populations identified in the B-GOS® and placebo groups, respectively (Additional file 4: Figure S3B; abundances above 1%).

Furthermore, *Bifidobacterium adolescentis* and *Bifidobacterium longum* were found to be the most abundant within *Bifidobacterium* spp., with *B*. *longum* being significantly predominant in ASD children under the exclusion diet compared to placebo (*P* < 0.02; Additional file 5: Figure S4).

### ^1^H-NMR analysis

#### Baseline

Orthogonal projections to latent structures-discriminant analysis (OPLS-DA) models were built to assess urinary and faecal metabolic perturbations induced by the different diets and B-GOS intervention. No significant metabolic differences were observed in the OPLS-DA model comparing urine spectra from ASD children on the two different diets (Q^2^ *Ŷ* = − 0.0199) at baseline (*N* = 60). Conversely, comparison of faecal profiles from children on the exclusion and unrestricted diets (*N* = 60) revealed distinct metabolic perturbations (Q^2^ *Ŷ* = 0.185, *P* < 0.001), at baseline, driven by the diets.

**Fig. 4:**
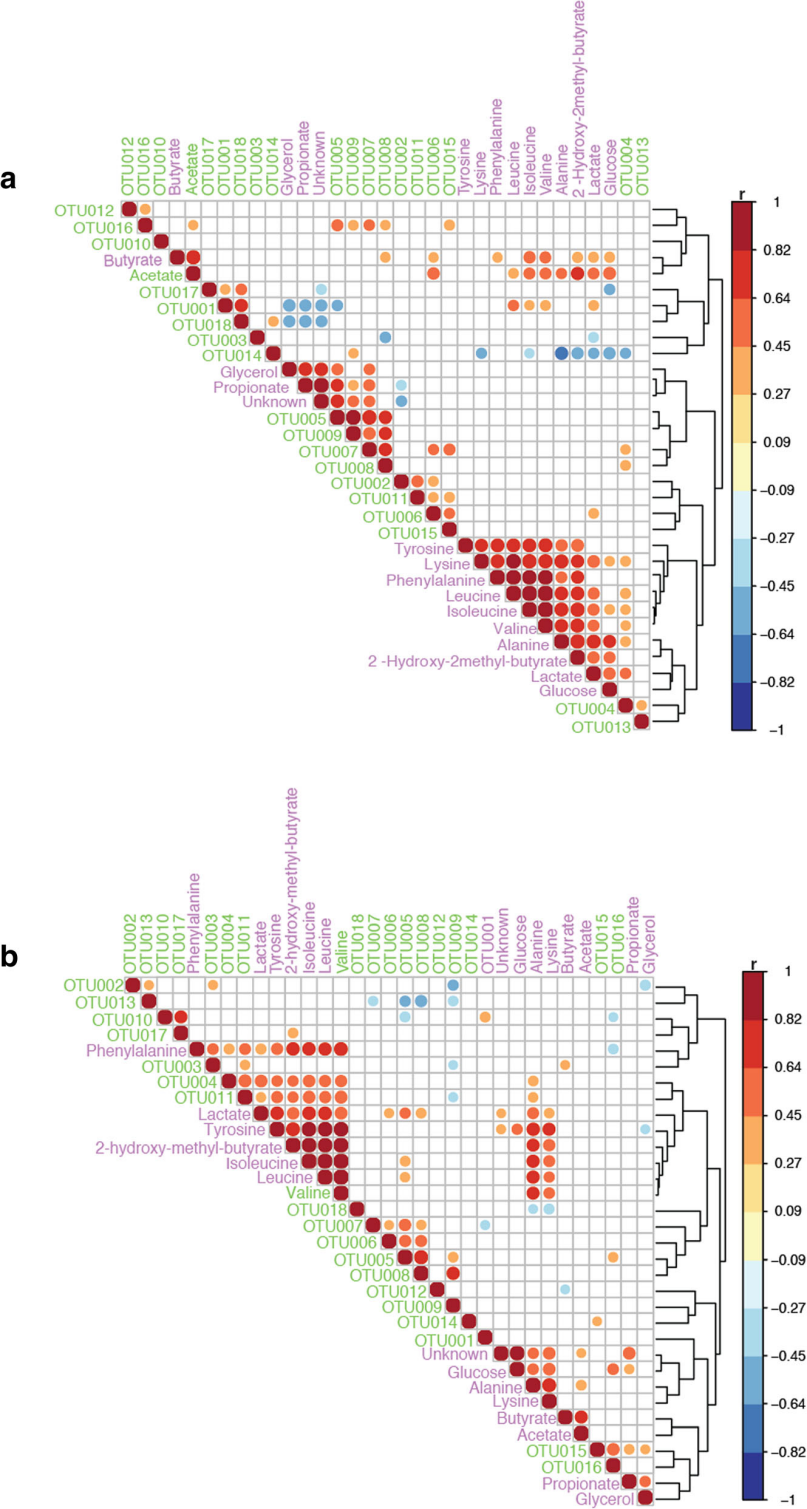
Summary of the correlation between bacterial changes and metabolic variation in faecal samples of children following exclusion diet **a** and those in un-restricted diet **b**. OUTs: bacterial groups. Arrows: metabolites identified; Squares: bacteria involved in the metabolic pathway. OTU001: *Bifidobacterium* spp.; OUT002: *Bifidobacterium longum*; OTU003: Coriobacteriaceae; OTU004: *Eggerthella lenta*; OTU005: *Bacteroides* spp.; OTU006: *B*. *fragilis*; OTU007: *B*. *ovatus*; OTU008: *B*. *uniformis*; OTU009: Rikenellaceae spp.; OTU010: *Lactococcus* spp.; OTU011: *Streptococcus arginosus*; OTU012: Clostridiales; OUT013: Clostridiaceae; OTU014: *Dehalobacterium* spp.; OTU015: *Roseburia* spp., OTU016: *F*. *prausnitzii*; OTU017: *Coprobacillus* spp.; OTU018: *Akkermansia muciphila*

Figure 4a, b summarise correlations between bacterial changes and metabolic variation in faecal samples of children following an exclusion diet (*N* = 24) and those on an unrestricted diet (*N* = 36), respectively. During the exclusion diet (Fig. 4a), *Bacteroides* spp. (OTU005, OTU007 and OTU008) strongly correlated with glycerol and propionate, whereas *Bifidobacterium* spp. (OTU001) and *Eghertella lenta* (OTU004) were positively correlated with valine, leucine and isoleucine. *E*. *lenta* was correlated to lysine and alanine. In faecal samples from children on the unrestricted diet (Fig. 4b), positive correlations were identified for *Eggerthella lenta* (OTU004) and *Streptococcus anginosus* (OTU011) with lactate, tyrosine, 2-hydroxy-2-methilbutyrate, isoleucine, leucine, phenylalanine and valine; for *Bacteroides* spp. (OTU006, TOU005 and OTU008) with lactate; for *F*. *prautznii* (OTU016) with glucose; and for *Coprobacillus* spp. (OTU017) with 2-hydroxy-2-methilbutyrate.

#### Intervention

Intervention with B-GOS® led to significant alterations in the urine spectra profiles of ASD children following unrestricted diets (*N* = 42), indicating that B-GOS® supplementation contributed to metabolic variation. A significant OPLS-DA model (Q^2^ *Ŷ* = 0.065; *R*^2^ *Ŷ* = 0.13; *P* < 0.001) was obtained by comparing metabolic profiles of ASD children taking placebo and those taking B-GOS®, after 6 weeks of intervention (Additional file 6: Figure S5). Urine spectra of autistic volunteers receiving B-GOS treatment contained greater amounts of creatinine, creatine, dimethylglycine (DMG), dimethylalanine (DMA), carnitine, citrate, adipate and trimethylamine-*N*-oxide (TMAO) compared to the autistic children taking placebo. In addition, B-GOS supplementation seemed to reduce amounts of phenylacetylglycine (PAG), phenylalanine and β-hydroxybutyrate in the test intervention group.

Metabolic shifts were also observed in faecal samples after B-GOS® intervention. At baseline (*N* = 28), a negative Q^2^ *Ŷ* was associated with ASD children on unrestricted diets (Q^2^ *Ŷ* = − 0.3632), but after B-GOS® supplementation (*N* = 42), a significant OPLS-DA model was obtained (Q^2^ *Ŷ* = 0.2997, *P* < 0.001). Ethanol, DMG and SCFAs (butyrate, valerate) were positively correlated with B-GOS® intake. Increases in butyrate production were also detected in ASD children following exclusion diets; however, these changes were not significant (data not shown). In addition, lower levels of amino acids (isoleucine, leucine, valine, alanine, glutamine) and lactate were detected in the B-GOS® group, compared to placebo (Additional file 7: Figure S6).

## Discussion

This is the first time that the impact of diet on GM composition and metabolism, their potential association with GI discomfort and the effect of a prebiotic supplementation has been investigated, in the context of autism. Significant differences, in both microbiota and metabolism, were detected at baseline when a comparison between the diets was performed. This could mean that diet has an impact on the gut environment. In addition, significant improvements in behavioural traits after B-GOS® supplementation, in ASD children following an exclusion diet, could indicate that combined interventions might be more beneficial in ASD individuals than a single dietary approach.

Before prebiotic B-GOS® intervention, we evaluated the nutritional impact of exclusion diets (GFCF) and our results showed deficiency in vitamin D intake, which was significant in children on unrestricted diets. These findings are supported by recent studies conducted in Spain, where an autistic group under a restricted diet (*n* = 105) consumed fewer carbohydrates (reported also in our study) and had higher consumption of legumes and vegetables [22, 23]. An interesting aspect from these studies, including ours, was the consistent identification of low vitamin D intake. Vitamin D is considered an active neurosteroid during brain development, and its deficiency has been suggested as a potential environmental risk factor for ASD [24–26]. Therefore, the association between ASD and Vitamin D deficiency warrants further investigation.

In addition to nutritional status, previous studies in ASD tended to evaluate the impact of exclusion diet on GI symptoms or behaviour, showing inconsistent results [27]. The hypothesis behind use of this diet is the so called ‘opioid excess theory’. This suggests that gluten and casein metabolism results in excessive production of opioid compounds causing side effects, such as constipation and behavioural problems [28]. Therefore, exclusion diets have been hypothesised to alleviate such issues but our study showed, in both restricted and unrestricted diets, that abdominal pain scores were below one. This means that GI dysfunctions were present but well tolerated in both groups, despite the significant difference observed, suggesting that the exclusion diet should not be considered a definitive solution for GI discomfort. Furthermore, recent randomised trials showed that ASD individuals tolerated gluten and casein introduction into their diet, questioning GFCF diet efficacy [29, 30].

Currently, metagenomic analyses in autism mainly focuses on identifying potential biomarkers in gut bacterial composition, usually comparing faecal samples from autistic individuals with siblings and non-autistic controls [31–33]. Results from these studies have identified several genera present in higher abundance in ASD, such as *Bacteroides*, *Roseburia*, *Akkermansia*, *Hespellia* spp., and others in lower abundance, such as *Prevotella* and bifidobacteria [34, 35].

There are no studies evaluating the effect of GFCF dietary approaches on GM in ASD individuals; therefore, our focus was to understand the impact of this diet on gut bacterial composition and metabolism. Our observations showed that volunteers following exclusion diets had lower abundances of bifidobacteria and Veillonellaceae, as previously reported in healthy adults [36]. These findings suggest that dietary restriction might have bigger impact on the growth of these bacterial groups, than type of disorder (e.g. autistic features).

In addition, metabolomic analyses showed amino acids (AA) to be the main metabolites present in faecal samples from both dietary groups, as seen previously [37]. This could be due to malabsorbtion of nutrients, since results obtained from the food diaries showed increase in protein and total sugar intake. These outcomes support the hypothesis that exclusion diet alone might not be enough to improve gut health.

Correlograms built in our analyses using bacterial composition and metabolites confirmed that bacterial groups such as *Clostridium* spp., *Bacillus* spp., *Lactobacillus* spp., and *Streptococcus* spp., are associated with AA metabolism [38]. It is known that AA are precursors for neurotransmitters, such as tryptophan for serotonin or tyrosine for catecholamines, but little is known on the impact of GM in these pathways and how diet might modulate it [39].

Our results also showed strong correlations between *Bacteroides* spp. and propionate in faecal samples of ASD children on exclusion diets. SCFAs have been shown to affect the CNS and in particular, intraventricular injection of propionate in mice has been seen to cause autistic like behaviour [40]. Our data show the potential role of the GM in the production of metabolites that might be associated with autistic traits. However, the actual impact of SCFAs produced in the gut on CNS needs to be further elucidated, since the concentrations that might pass through the blood-brain barrier (BBB) may be small.

Overall, our data following B-GOS® intervention did not show a significant impact on GI symptoms and sleep, even though a trend of reduction of GI discomforts was observed (data not shown). The reason for this might in part be due to various difficulties reported by the parents in evaluating these aspects and related to impediments in communication skills that are typical of ASD children.

Combining B-GOS® treatment with the exclusion diet showed a significant reduction in anti-sociability scores, supporting the hypothesis that combined intervention therapies might have a better impact on such psychological traits. This is the first study to show a synergistic effect between exclusion diet and a prebiotic intervention and the results are promising. Interestingly, even though no significant differences were reported over time between baseline and prebiotic intervention in this group (data not shown), significant changes were detected in the gut microbiota composition of ASD children following exclusion diet when variable ‘treatment’ (placebo against B-GOS®) was analysed after 6 weeks of intervention. In addition, in this intervention group (exclusion diet + B-GOS®), significantly higher abundance of *B*. *longum* was observed, matching previous results reported in a recent human intervention study [41], where administration of *B*. *longum* 1714 reduced stress and improved memory [41]. These results strengthen the potential beneficial role of these bacteria on CNS and impact of these combined dietary approaches.

To our knowledge, this is the first study where 16S rRNA gene amplicon sequencing has been used to better understand the impact of a prebiotic intervention on GM in autism. B-GOS was able to modulate the GM composition in autistic children on unrestricted diets, modulating bifidobacterial changes as well as in other bacterial groups, such as Lachnospiraceae family (*Coprococcus* spp., *Dorea formicigenerans*, *Oribacterium* spp.), known to be butyrate-producing bacteria. Mego and colleagues reported the same results in a human intervention study in healthy adults where regular consumption of B-GOS® treatment-induced changes in the gut microbiota, and its modulation was correlated with reduced gas production [42].

We supported these results by ^1^H-NMR metabolomics, detecting butyrate as main SCFAs produced and matching our previous in vitro data, where B-GOS supplementation modulated bacterial and metabolic changes in ASD [21]. It has been shown, in cell culture studies, that butyrate regulates tyrosine hydroxylase (TH) mRNA levels and consequently may affect catecholamine pathway in the brain [43–47], thus potentially positively impacting ASD [48]. Therefore, showing that B-GOS was able to stimulate butyrate production in ASD in vitro and in vivo suggests that it could have an indirect effect on the CNS through modulation of gut bacterial populations.

Interestingly, we also observed a reduction of AA in faecal samples of ASD children taking B-GOS®. The presence of these compounds in faeces has been previously associated with problems in gut barrier function which could lead to malabsobtion of dietary components, typical of disorders related to gut inflammation, such as Inflammatory Bowel Disease (IBD) [49]. Therefore, decreasing AA excretion suggests that B-GOS® supplementation could help to improve gut health. This could be associated with butyrate production, which increased after B-GOS® intervention, since improvements in barrier functions have been previously detected in vitro with sodium butyrate [50].

Some confounding factors might impact the analysis of urine samples, such as medication, diet and lifestyle. These are difficult to control, especially when the sample size is small; even though diet and medication intake were recorded during the study. Significant correlations were identified in ASD children under unrestricted diets between citrate, creatine, creatinine and B-GOS®. Inconsistent levels of creatinine have been detected in ASD individuals and are considered as potential biomarkers for creatine deficiency syndrome (CDS), a metabolic disorder with similar features to autism [51]. In addition, they have been considered as biomarkers for other brain disorders such as Alzheimer’s disease [52] and schizophrenia [53], so it would be interesting to have a deeper understanding of implications for ASD.

## Conclusions

In summary, this was the first trial to assess the impact of exclusion diets and a prebiotic intervention on GM composition and metabolic activity in ASD. The study showed a significant impact of diets on GM. An exclusion diet resulted in a high AA excretion and potential problem in nutrient malabsorption, suggesting that it should be reconsidered as first dietary intervention to improve gut issues. A combined dietary approach of a prebiotic and exclusion diet, resulted in a significant improvement in antisocial behaviour suggesting that such approaches might be more relevant for improvement of these aspects as well as psychological traits.

## Methods

### Study design

A randomised, double-blind, placebo-controlled (Maltodextrin—GLUCIDEX®; 1.8 g), parallel-designed prebiotic B-GOS® mixture (Bimuno®; 1.8 g: 80% GOS content) feeding study was conducted in children diagnosed with ASD. Both treatments were provided in powder form and supplied by Clasado Biosciences Ltd. (Reading, UK). The study was conducted according to the guidelines of the Declaration of Helsinki, and the University of Reading Research Ethics Committee (UREC15/41) approved all procedures involving human subjects.

### Subjects

**Fig. 5:**
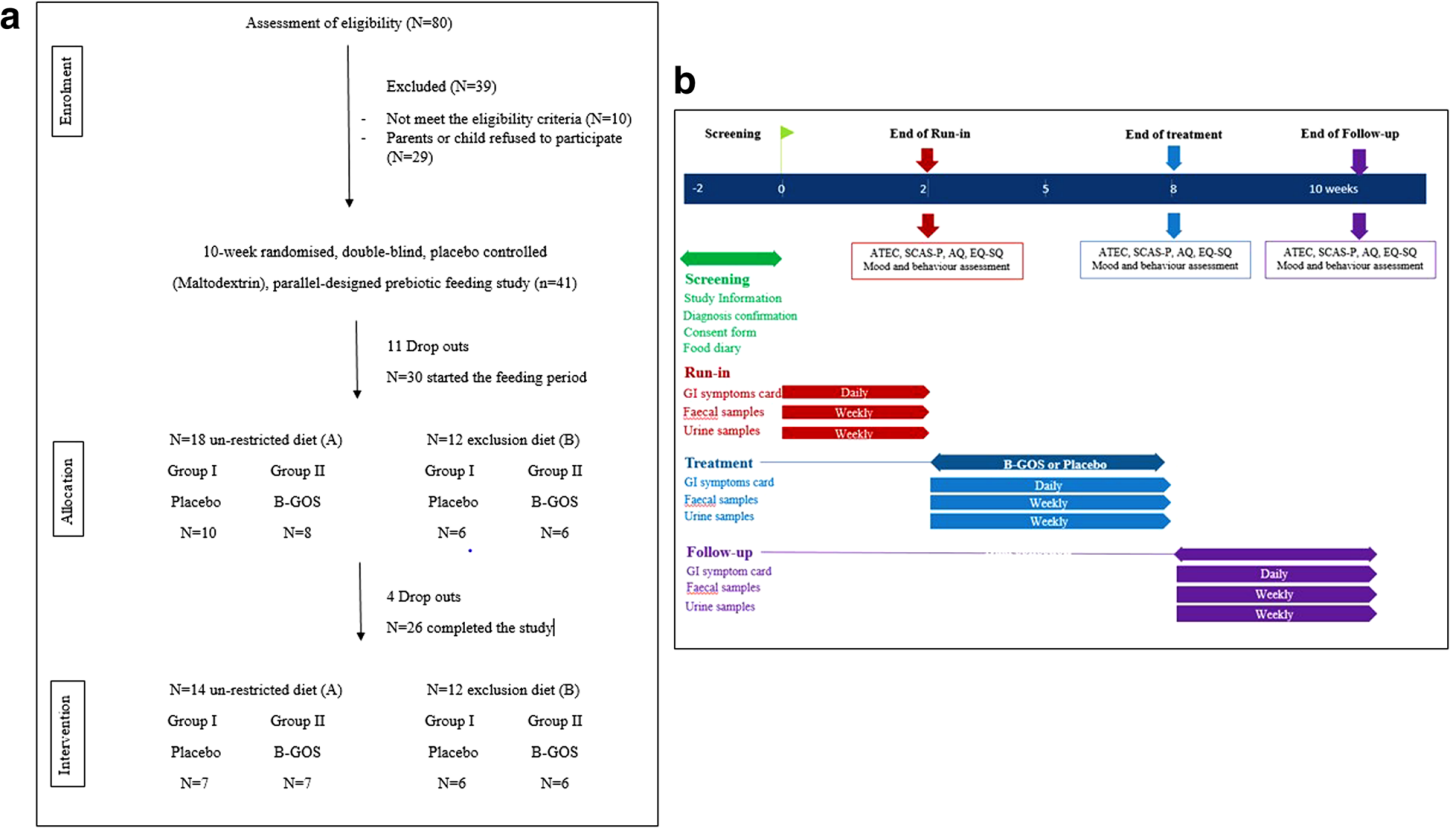
Study information. **a** Flow of participants through the study. **b** Study design

A total of 41 autistic children (31 male and 10 female; mean age 7.7 years old; range 4–11 years) with formal diagnosis of ASD were enrolled in the study (Fig. 5) in order to have a 95% probability that the study would detect, at a two-sided 5% significance level, a significant effect on the colonic bifidobacterial population. Of these 41, 11 subjects withdrew from the study during the baseline period, largely due to difficulties and/or inconveniences associated with collecting samples. The remaining 30 volunteers were divided into two groups A and B, according to dietary habits assessed by 4-day food diaries, children whose diet was not restricted (*n* = 18) and on exclusion diet (*n* = 12), mainly gluten and casein free. Within these groups, children were randomly assigned to two feeding groups using a random number system. Group I received placebo and group II received B-GOS® during the 6-week feeding period. However, four subjects subsequently dropped out before the end of the first feeding arm. One left the study without giving a reason, two were withdrawn due to protocol violation, and one withdrew due to an adverse event (strong diarrhoea and abdominal pain observed after 2 days of treatment feeding). In total, 26 volunteers completed the 10-week study providing 80% statistical power. Volunteers were assessed before the start of the trial and were selected according to certain exclusion and inclusion criteria. Inclusion criteria for participation in the study were signed consent form from volunteers’ parents or guardians, age of 4–11 years inclusive, formal diagnosis of ASD (severity was not taken into consideration; Table 2). Exclusion criteria involved the use of probiotics, prebiotics, antibiotics, or other dietary supplement drugs that could affect the luminal microenvironment of the intestine, within 4 weeks before the study, were also excluded. Volunteers were instructed not to consume such products during the study and not to alter their usual diet or fluid intake. Volunteers were required to visit the University of Reading or the researcher visited the volunteers at home on five separate occasions during the study period in order to provide faecal and urine samples (weekly collection).

**Table 2:** Diagnosis (*) reported from parents by medical assessment

Diagnosis*	ASD	ADHD	Asperger	PDD
Volunteers (*n*)	26	5	1	1
Exclusion diet B-GOS (B-II)	6			1
Exclusion diet Placebo (B-I)	6	3	1	
Un-restricted diet B-GOS (A-II)	7	1		
Un-restricted diet Placebo (A-I)	7	1		

All participants were diagnosed with ASD (*n* = 26) and some volunteers also had additional diagnoses (ADHD, Asperger, PDD)

*ASD* autism spectrum disorders, *ADHD* attention deficit hyperactivity disorder, *PDD* pervasive development disorder

### Dietary intervention and assessments

Food diary records were kept for four consecutive days during the baseline period. Before starting with the intervention, food diaries were discussed with parents in order to monitor compliance. At the front of the diary, detailed information on how to record food and beverages consumed using common household measures were provided. Food diaries were analysed using Diet-plan7 software (Forestfield Software Ltd.)

### GI symptoms

Parents/guardians of child volunteers were asked to fill in daily questionnaires for GI function and symptoms. The Bristol stool chart was used to assess faecal sample type and consistency, together with the number of bowel movements, abdominal pain, intestinal bloating and flatulence [54]. Concomitant medication, adverse events, changes in diet and behaviour were also recorded throughout the study, on separate sample submission forms.

### Behavioural and sleep assessment

Parents/guardians were asked to complete an Autism Treatment Evaluation Checklist (ATEC; [55]) to evaluate effectiveness of treatment; autism spectrum quotient (AQ; [56]) in order to assess the autism symptoms; empathy and systemising quotient (EQ-SQ; [57]) to assess the capacity of the child to understand emotions and thoughts and how to process them; and Spence’s Children Anxiety Scale-Parent version (SCAS-P; [58]) in order to identify levels of anxiety. In addition, parents/guardians were asked to complete 5-day sleep diaries before (baseline) and after intervention (during last week of the intervention period) in order to understand the qualitative impact of B-GOS supplementation on sleep disorder, if present.

### Faecal sample collection and preparation

Faecal samples were collected once a week using a faecal collection kit (FC2040, Laboratories Ltd., UK), and volunteers were asked to keep them at − 20 °C until the visit day, when they were transferred to the laboratory and processed. Samples for DNA extraction and ^1^H-NMR were weighed (~ 250 mg) and stored at − 80 °C until needed for analysis.

### Urine sample collection and preparation

Urine samples were collected once a week using sterile tubes (Mid-Stream Urine Specimen Collector; Pennine Healthcare, UK) or sterile pads (Sterisets Urine Collection Kit; MediBargains, UK) and stored at − 20 °C until a visit day when they were processed. Samples were transferred to 15-ml falcon tubes, centrifuged for 10 min at 1136×*g*, supernatant transferred to 1.5-ml Eppendorf tube (duplicate) and stored at − 80 °C.

### DNA extraction

Total microbial DNA was extracted from faeces using the DNA stool mini kit (Qiagen, UK) by introducing three 1-min steps at 50 movements/s using TyssueLyser LT (Qiagen, UK) with 5-min incubation in ice between treatments as previously described by Candela et al. 2016 [59]. DNA recovery was evaluated using a NanoDrop ND-1000 spectrophotometer (NanoDrop Technologies).

### Fluorescence in situ hybridisation (FISH)

Synthetic oligonucleotide probes targeting specific regions of the 16S rRNA labelled with the fluorescent dye Cy3, as previously described by Grimaldi et al. 2016 [60], were used for bacterial enumeration assessed by FISH analysis. The probes used (Eurofins Genomics, UK) were Bif164 for *Bifidobacterium* spp*.* [61] and EUB338 I-II-III [62]. EUB338 I-II-III probe was used to calculate total bacterial numbers. This value was multiplied by the abundances (%) of each bacterial group obtained from the 16S sequencing analysis and then converted in Log_10_. Bif164 probe has been used to evaluate the bifidogenic activity of the prebiotic product due to its high specificity for bifidobacterial group.

### 16S rRNA gene amplification via next-generation sequencing (NGS) and bioinformatics analysis

For each sample, the V3–V4 region of the 16S rRNA gene was PCR-amplified in 25 μl volumes containing 12.5 ng of microbial DNA, 2× KAPA HiFi HotStart ReadyMix (Kapa Biosystems, USA) and 200 nmol/l of S-D-Bact-0341-b-192S-17/ S-D-Bact-0785-a-A-21 primers carrying Illumina overhang adapter sequences (Bio-Fab Research). Thermal cycling consisted of an initial denaturation at 95 °C for 3 min, 25 cycles of denaturation at 95 °C for 30 s, annealing at 55 °C for 30 s, extension at 72 °C for 30 s and a final extension step at 72 °C for 5 min. Amplicons of 440 bp were purified with a magnetic bead-based clean-up system (Agencourt AMPure XP; Beckman Coulter) and sequenced on Illumina MiSeq platform using a 2 × 250 bp paired end protocol, according to the manufacturer’s instructions (Illumina, San Diego, CA). Libraries were pooled at equimolar concentrations, denatured and diluted to 4 nmol/l. Pair-ended amplicon reads (with corresponding quality scores) were trimmed, merged, clustered (operational taxonomic units [OTU] with 97% similarity), filtered from chimeric sequences using UPARSE [63] and taxonomically assigned using the GreenGenes database (version 12.10) [64]. For downstream analysis, the OTU-table was normalised with cumulative sum scaling (CSS) [65] using the Qiime toolbox (*v*1.9) [66]. Data from volunteers were pooled together during the analysis, and only the samples at baseline (weeks 1 and 2), after treatment (weeks 6, 7 and 8) and follow up (weeks 9 and 10) were considered for the analysis. Beta-diversity was assessed through Bray-Curtis and Søresen-Dice distances and the factor-treatment analysed with redundancy analysis (RDA) [67]. Alpha-diversity was measured and expressed as observed species (97% similarity OTUs) and computed with 10 rarefied OTU tables. Comparison of Alpha-diversities was made through nonparametric *t*-test method (Monte Carlo, 999 permutations).

### Metabolic analysis by ^1^H-NMR

Four hundred microliters of urine samples were combined with 200 μL of phosphate buffer [0.2 M (pH 7.4) in D2O plus 0.001% TSP (3-(trimethylsilyl)-[2,2,3,3,−2H4]-propionic acid, δ 0.00)], mixed by vortexing, centrifuged at 1136×*g* for 10 min, and then 550 μL was transferred into 5 mm NMR tubes for analysis. Faecal samples were pre-weighed (250 mg) and 700 μL of phosphate buffer and 2 glass beads added in order to perform a bead-beating 5 min step at 25 movements/s using TyssueLyser LT (Qiagen, UK). Then, 500 μL was transferred into 5-mm NMR tubes for analysis. All NMR spectra were acquired on a Bruker Avance DRX 500 MHz NMR spectrometer (Bruker Biopsin, Rheinstetten, Germany) operating at 500 MHz. They were acquired using a standard one-dimensional (1D) pulse sequence [recycle delay (RD)–90°–t1–90°–tm–90°-acquire free induction decay (FID)] with water suppression applied during RD of 2 s, a mixing time Tm of 100 ms and a 90 pulse set at 7.70 μs. For each spectrum, a total of 128 scans were accumulated into 64 k data points with a spectral width of 12.001 ppm. The FIDs were multiplied by an exponential function corresponding to 0.5 Hz line broadening.

### Data preprocessing and analysis

Data from volunteers were pooled together during the analysis. Samples at baseline (weeks 1 and week 2), after treatment (weeks 6, 7 and 8) and follow-up (weeks 9 and 10) were considered for the analysis. All spectra were manually phased, baseline corrected and calibrated to the chemical shift of TSP using TopSpin (Bruker Biopsin, Rheinstetten, Germany). Spectra were digitised using an in-house MATLAB (version R2014a, The Mathworks, Inc.; Natwick, MA, USA), and median fold normalisation was performed. The spectral region containing the water resonance was removed to minimise distortions in the baseline arising from imperfect water saturation. Principal component analysis (PCA) using mean-centred data was applied and orthogonal projection to latent structure discriminant analysis (OPLS-DA) models were constructed using for pairwise comparisons of the different experimental groups and time points. Colour represents the significance of correlation (*r*) for each metabolite to class membership. Predictive strength (Q^2^ Ŷ) of the models was obtained using a sevenfold cross-validation method and validated using permutation testing (number of permutations = 1000).

### Statistical analysis

Behavioural assessments and GI symptoms were analysed using SPSS software v.22 (SPSS Inc., Chicago, IL, USA). Mood and behavioural questionnaire analyses were performed considering the single scale scores and total score, for each time point (before T1, after treatment T2 and follow-up T3). One-way ANCOVA was used in order to test whether T2 scores differed between treatment groups, adjusting for baseline T1 scores as covariate. GLM 2 × 2 × 3 RMANOVAs were used to test whether there were any diet by treatment by time interactions across time points. Each of those RMANOVAs was followed by a 2 × 2 × 2 RMANCOVA to test for any diet by treatment by time interactions across T2 and T3 adjusting for baseline (T1) differences (post hoc tests are not valid where covariates are included); age was included as a covariate when significant since some assessments may vary by age. This test was used in order to assess whether any treatment effect was consistent. GI symptoms were analysed using a linear mixed model. The fixed terms assessed in this model were diet, treatment, time (pre-treatment, during treatment, follow-up), weeks, diet × time, treatment × time and volunteer scores as a random effect.

Statistical test for food intake records was performed using Graphad Prism (version 5.0; Graph-Pad Software, 188 La Jolla, CA, USA). Normality test was used to assess whether the data were parametric or not parametric and unpaired Student’s *t* test and Mann-Whitney tests (Bonferroni post-test with significance set at *P* < 0·05) were performed respectively on food diary data set in order to assess statistical differences between exclusion and un-restricted diets.

## Additional files

Additional file 1: **Figure S1.** Comparison of the gut microbiota composition between ASD children following exclusion diet and ASD children following unrestricted diet. (A) Sørensen-Dice distance based PCoA; (B) Bray-Curtis distance based PCoA. Red dots: exclusion diet; green dots: unrestricted diet. (JPG 51 kb)

Additional file 2: **Table S1.** Bacterial groups (Log10) significantly associated with separation in RDA model (*P* < 0.004). (DOCX 15 kb)

Additional file 3: **Figure S2.** Comparison of bacterial richness and diversity before and after B-GOS treatment in ASD children following unrestricted diet. Rarefaction curves and box plots showed that B-GOS supplementation increased the diversity in gut microbial composition of ASD children in unrestricted diet. (JPG 83 kb)

Additional file 4: **Figure S3.** (A) RDA model showing the separation between samples from ASD children following exclusion diet after the intervention (placebo vs B-GOS®). Blue dots: samples from children taking placebo; Pink dots: samples from children taking B-GOS®. (B) Bar chart of the most abundant bacteria in ASD children following exclusion diet after intervention (placebo vs B-GOS®; bacterial abundances above 1%). (JPG 174 kb)

Additional file 5: **Figure S4.** Analysis of the most abundant *Bifidobacterium* spp. using 16S rRNA sequencing. (1) before B-GOS®; (2) after B-GOS®; (3) before placebo; (4) after placebo; (5) unrestricted diet before B-GOS®; (6) unrestricted diet after B-GOS®; (7) unrestricted diet before placebo; (8) unrestricted diet after placebo; (9) exclusion diet before B-GOS®; (10) exclusion diet after B-GOS®; (11) exclusion diet before placebo; (12) exclusion diet after placebo. *P < 0.05. (JPG 65 kb)

Additional file 6: **Figure S5.** OPLS-DA obtained comparing the metabolic profile in urine samples of ASD children in unrestricted diet taking B-GOS® to those taking placebo. Compounds identified: dimethylglycine (DMG); dimenthylalanine (DMA); creatinine; creatine; PAG (Phenylacetilglycine); cartine; malonate; TMAO (trimethylamine-*N*-oxide); citrate; adipate; beta-hydroxybutyrate; phenylalanine. (JPG 79 kb)

Additional file 7: **Figure S6.** OPLS-DA obtained comparing the metabolic profile in faecal samples of ASD children in unrestricted diet taking B-GOS to those taking placebo. Compounds identified: dimethylglycine (DMG); glutamate; butyrate; valerate; ethanol; alanine; lactate; isoleucine; leucine; valine; uracil; phenylalanine; tyrosine. (PNG 100 kb)
