# Supplementary figures and images for: A prebiotic intervention study in children with autism spectrum disorders (ASDs)
Source: Microbiome. 2018 Aug 2;6:133. doi: 10.1186/s40168-018-0523-3 (PMC6091020; doi:10.1186/s40168-018-0523-3)

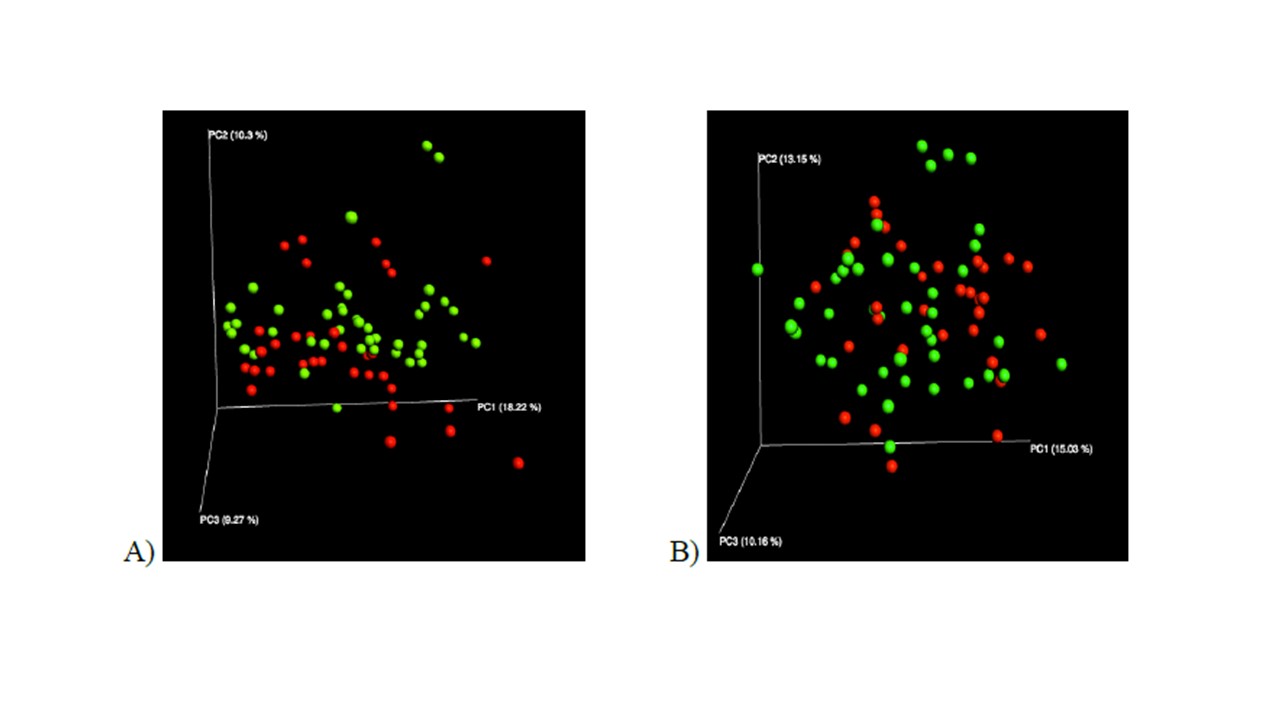

Supplement: Supplementary file 1 — Figure S1. Comparison of the gut microbiota composition between ASD children following exclusion diet and ASD children following unrestricted diet. (A) Sørensen-Dice distance based PCoA; (B) Bray-Curtis distance based PCoA. Red dots: exclusion diet; green dots: unrestricted diet. (JPG 51 kb) [file 40168_2018_523_MOESM1_ESM.jpg]

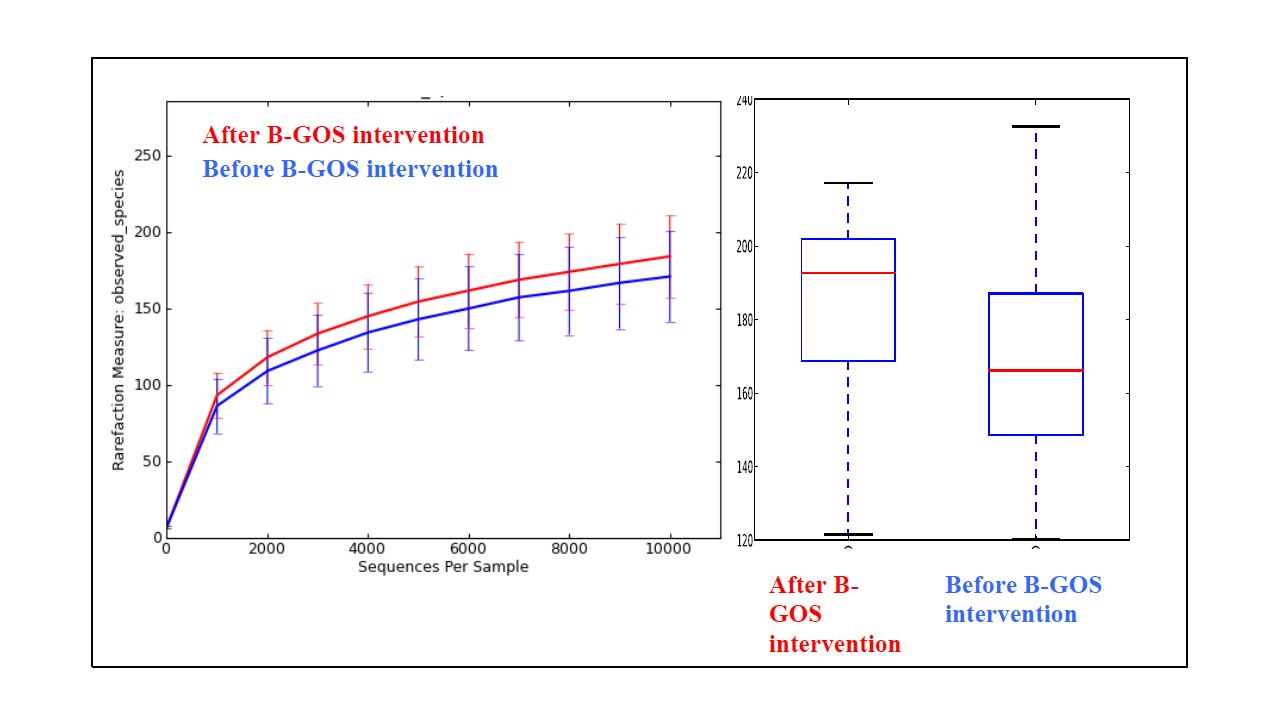

Supplement: Supplementary file 3 — Figure S2. Comparison of bacterial richness and diversity before and after B-GOS treatment in ASD children following unrestricted diet. Rarefaction curves and box plots showed that B-GOS supplementation increased the diversity in gut microbial composition of ASD children in unrestricted diet. (JPG 83 kb) [file 40168_2018_523_MOESM3_ESM.jpg]

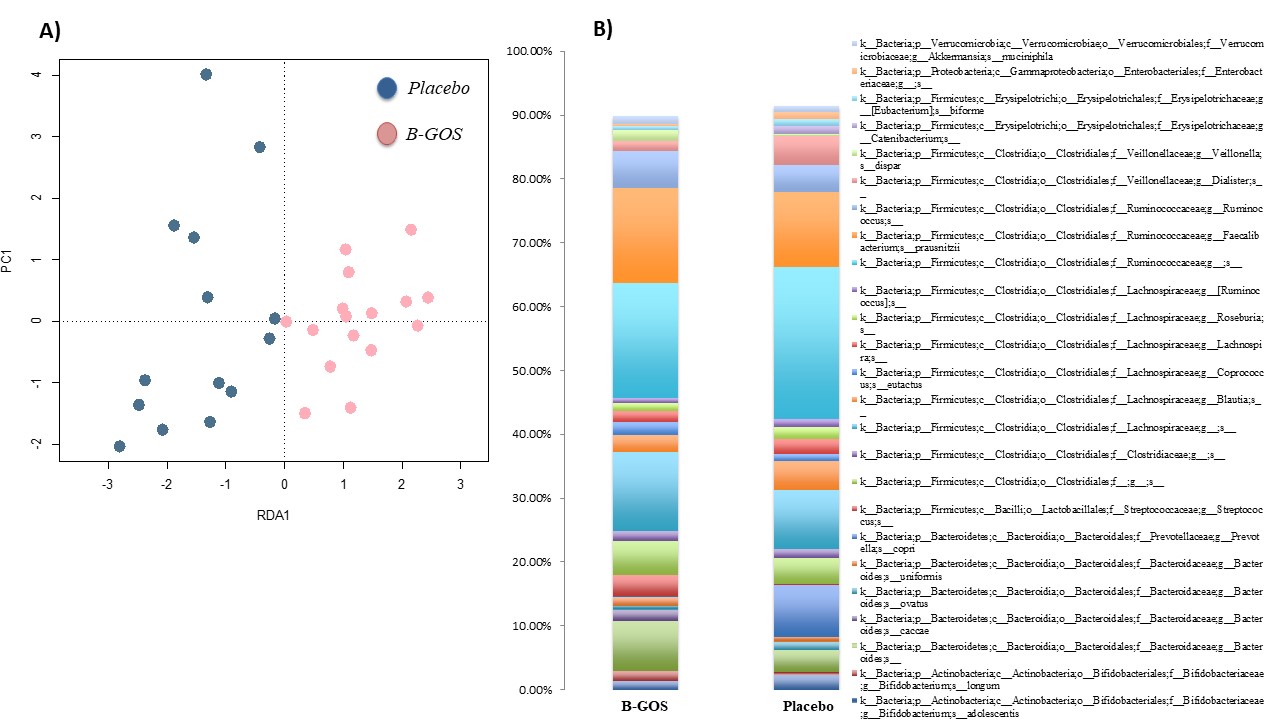

Supplement: Supplementary file 4 — Figure S3. (A) RDA model showing the separation between samples from ASD children following exclusion diet after the intervention (placebo vs B-GOS®). Blue dots: samples from children taking placebo; Pink dots: samples from children taking B-GOS®. (B) Bar chart of the most abundant bacteria in ASD children following exclusion diet after intervention (placebo vs B-GOS®; bacterial abundances above 1%). (JPG 174 kb) [file 40168_2018_523_MOESM4_ESM.jpg]

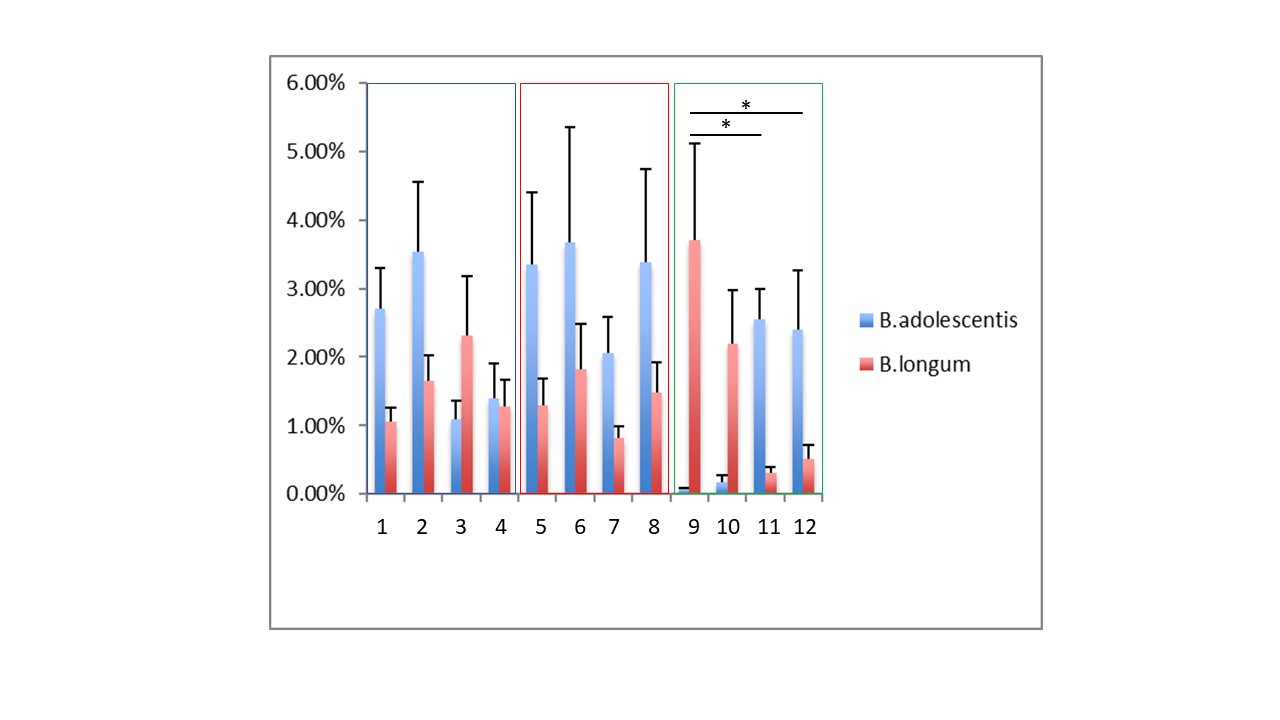

Supplement: Supplementary file 5 — Figure S4. Analysis of the most abundant Bifidobacterium spp. using 16S rRNA sequencing. (1) before B-GOS®; (2) after B-GOS®; (3) before placebo; (4) after placebo; (5) unrestricted diet before B-GOS®; (6) unrestricted diet after B-GOS®; (7) unrestricted diet before placebo; (8) unrestricted diet after placebo; (9) exclusion diet before B-GOS®; (10) exclusion diet after B-GOS®; (11) exclusion diet before placebo; (12) exclusion diet after placebo. *P < 0.05. (JPG 65 kb) [file 40168_2018_523_MOESM5_ESM.jpg]

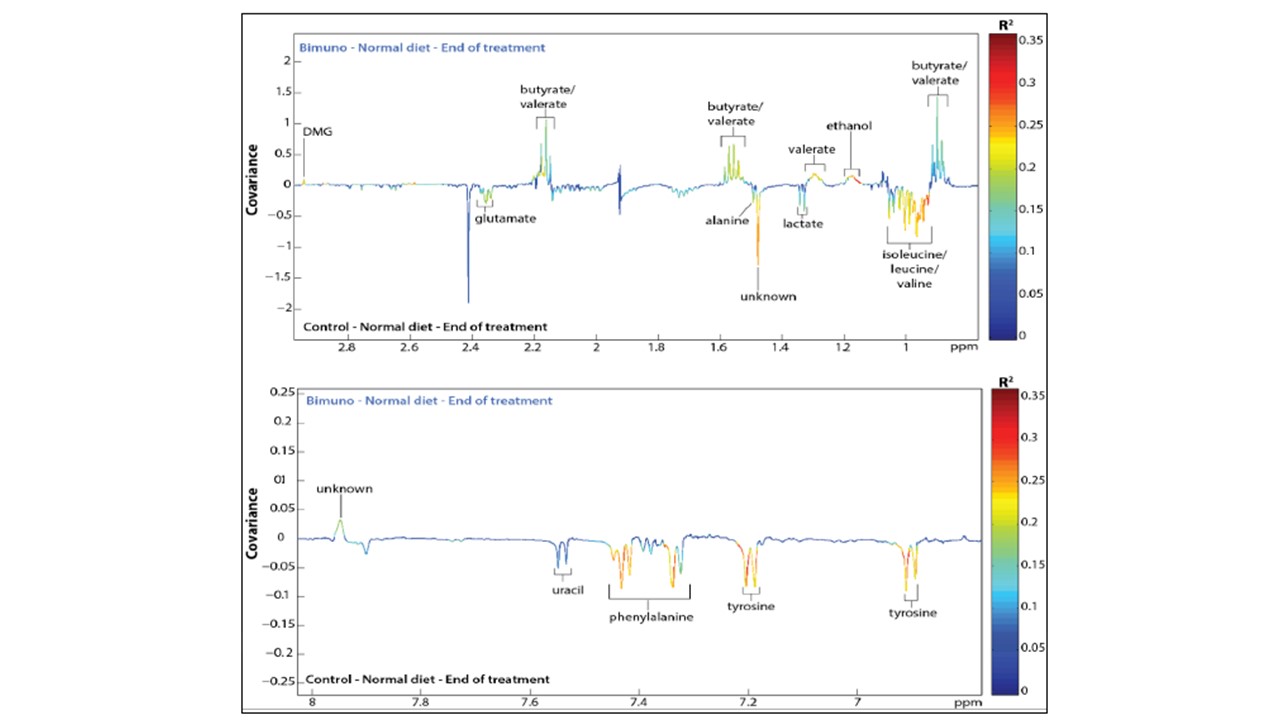

Supplement: Supplementary file 6 — Figure S5. OPLS-DA obtained comparing the metabolic profile in urine samples of ASD children in unrestricted diet taking B-GOS® to those taking placebo. Compounds identified: dimethylglycine (DMG); dimenthylalanine (DMA); creatinine; creatine; PAG (Phenylacetilglycine); cartine; malonate; TMAO (trimethylamine-N-oxide); citrate; adipate; beta-hydroxybutyrate; phenylalanine. (JPG 79 kb) [file 40168_2018_523_MOESM6_ESM.jpg]

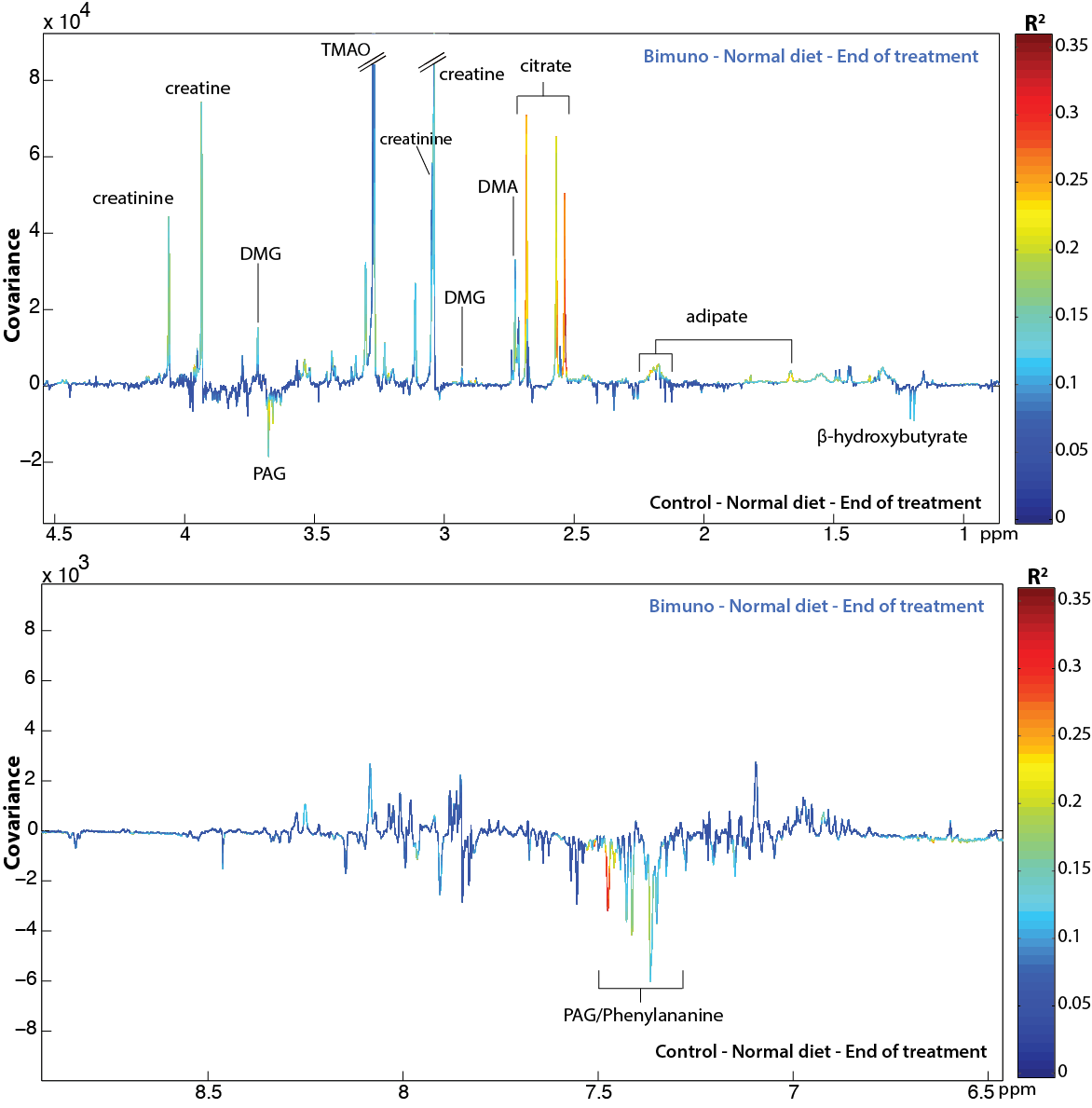

Supplement: Supplementary file 7 — Figure S6. OPLS-DA obtained comparing the metabolic profile in faecal samples of ASD children in unrestricted diet taking B-GOS to those taking placebo. Compounds identified: dimethylglycine (DMG); glutamate; butyrate; valerate; ethanol; alanine; lactate; isoleucine; leucine; valine; uracil; phenylalanine; tyrosine. (PNG 100 kb) [file 40168_2018_523_MOESM7_ESM.png]
