# Supplementary material for: A prebiotic intervention study in children with autism spectrum disorders (ASDs)
Source: Microbiome. 2018 Aug 2;6:133. doi: 10.1186/s40168-018-0523-3 (PMC6091020; doi:10.1186/s40168-018-0523-3)
Supplement: Supplementary file 2 — Table S1. Bacterial groups (Log10) significantly associated with separation in RDA model (P < 0.004). (DOCX 15 kb) [file 40168_2018_523_MOESM2_ESM.docx]

**Table S1: bacterial groups (Log10) significantly associated with separation in RDA model (P<0.004)**

| Bacterial groups | Un-restricted diet | Exclusion diet |
| --- | --- | --- |
| *Bifidobacterium* spp | 9.25 | 9.09 |
| Coriobacteriaceae | 8.99 | 8.26 |
| *Eggerthella lenta* | 8.06 | 7.53 |
| *Bacteroides fragilis* | 9.33 | 8.85 |
| *Lactococcus* spp | 7.74 | 6.11 |
| *Streptococcus anginosus* | 7.05 | 6.20 |
| Clostridiaceae | 8.74 | 8.03 |
| *Coprobacillus* spp | 8.22 | 7.58 |
| *Akkermansia muciniphila* | 10.13 | 9.59 |
| *Bacteroides* spp | 9.78 | 10.06 |
| *Bacteroides ovatus* | 9.06 | 9.20 |
| *Bacteroides uniformis* | 9.40 | 9.38 |
| Rikenellaceae | 9.00 | 8.92 |
| *Dehalobacterium* spp | 6.53 | 7.22 |
| *Roseburia* spp | 9.23 | 9.48 |
| *Faecalibacterium prausnitzii* | 10.32 | 10.40 |
